# Development of novel SARS-CoV-2 viral vectors

**DOI:** 10.1038/s41598-023-40370-8

**Published:** 2023-08-11

**Authors:** Huan Liu, Dexi Liu

**Affiliations:** Department of Pharmaceutical and Biomedical Sciences, College of Pharmacy, University of Georgia, 250 West Green Street, Athens, GA 30602 USA

**Keywords:** Biological techniques, Microbiology

## Abstract

The authentic SARS-CoV-2 requires to be handled in Biosafety Level 3 laboratories, which restrains investigation by the broader scientific community. Here, we report the development of a novel SARS-CoV-2 viral vector composed of all 4 SARS-CoV-2 structural proteins, the packaging signal sequence of SARS-CoV-2, a reporter gene, and an RNA amplification component of Venezuelan equine encephalitis virus (VEEV). This VEE-SARS-CoV-2 viral vector transduces target cells in an ACE2-dependent manner, and all 4 structural proteins of SARS-CoV-2 are indispensable for its transduction activity. Comparative studies show that the incorporation of the VEEV self-amplification mechanism increases the gene expression level by ~ 65-fold and extends the transgene expression up to 11 days in transduced cells. Additionally, we demonstrated the significant applications of this new VEE-SARS-CoV-2 vector for neutralizing antibody quantification and antiviral drug testing. The VEE-SARS-CoV-2 vectors developed will be an important and versatile tool for investigating SARS-CoV-2 molecular virology, developing antiviral agents targeting receptor binding, and studying RNA genome packaging and function of the essential but not well studied structural proteins of SARS-CoV-2.

## Introduction

Severe acute respiratory syndrome coronavirus 2 (SARS-CoV-2), the causative agent of coronavirus disease 2019 (COVID-19), has posed unparalleled threat to global public health. As a family member of *Coronaviridae*, SARS-CoV-2 has 4 structural proteins: the spike (S) protein, envelop (E) protein, membrane (M) protein, and nucleocapsid (N) protein bound to its ~ 30 kb positive-strand RNA genome. SARS-CoV-2 S protein binds to human angiotensin-converting enzyme 2 (ACE2) and is activated by cellular proteases such as transmembrane protease serine 2 (TMPRSS2) to enter its host cells^1,2^. Significant efforts have been made to dissect molecular virology of SARS-CoV-2 and develop various strategies to block viral infection by vaccination or antiviral drugs^3,4^. Nevertheless, research involving the highly infectious SARS-CoV-2 requires BSL-3 conditions to contain its virulence, and the limited access to BSL-3 laboratories has been a major barrier to combat against this virus.

To enable SASR-CoV-2 research in most laboratories, various model systems have been developed to substitute the wild type SARS-CoV-2. In SARS-CoV-2 reporter replicon systems, spike protein is replaced with either green fluorescence protein (GFP) or luciferase (Luc) reporter to screen drugs targeting viral RNA replication^5–8^. A self-propagating hybrid replicon system utilizes VEEV replicon to express spike protein and induce cell-to-cell transmitted syncytia formation^9^. Lentiviral and vesicular stomatitis viral vectors have been incorporated with SARS-CoV-2 spike protein to confer the virion SARS-CoV-2 tropism, thereby suitable for assessment of neutralizing antibodies and antiviral compounds^10,11^. However, replicons and pseudotyped viruses developed so far lack one or more structural proteins of SARS-CoV-2 and fail to recapitulate the critical steps of SARS-CoV-2 life cycle, such as viral assembly and egress. An alternative strategy utilizes virus-like particles (VLPs) to model virus life cycle in cellular context through co-transfection of plasmids encoding SARS-CoV-2 structural protein genes (S, E, M, N). SARS-CoV-2 VLPs have been made in mammalian cells^12–15^, plant cells^16^, and insect cells^17^. Those VLP systems are useful for modeling viral budding, particle formation, and entry into host cells, yet they lack the ability to package any gene of interest for quantitation purpose. This problem was solved by Syed et al. establishing a new type of SARS-CoV-2 virus-like particles (SC2-VLPs) capable of packaging and delivering exogeneous transgene into ACE2 + cells^18^. The SC2-VLP system was used to examine the effect of structural gene mutations on viral fitness and infectivity of evolved variants^19^. The SC2-VLP system has unique advantages over other model systems, but its broader application is limited by the low gene expression level and short duration of transgene expression.

In this study, we developed a novel SARS-CoV-2 viral vector composed of the 4 SARS-CoV-2 structural proteins (S, E, M, N) carrying a self-amplifying VEEV replicon, a putative SARS-CoV-2 packaging signal, and a reporter gene. The self-amplifying replicon composed of genes encoding VEEV non-structural proteins 1–4 (nsp1-4) was incorporated to form an RNA-dependent RNA polymerase (RdRP) complex to enhance transgene expression by transcript amplification. VEEV replicon was selected because it is a single positive-stranded RNA without activity of genome-integrating^20^; it can generate high copy number of transgene and extended gene expression^21,22^; and its safety profile has been demonstrated by ongoing preclinical and clinical trials for COVID-19 vaccine development^23–25^.

The developed VEE-SARS-CoV-2 vectors have unique advantages over other systems: its superior safety profile allowing researchers to use in BSL-2 laboratories; the structural resemblance to wild type SARS-CoV-2 for modeling viral infection; and the significantly higher and extended gene expression with a wider quantitation range. We also demonstrate that our vector system can be used for neutralizing antibody titration and antiviral drug screening. The VEEV-SARS-CoV-2 system developed provides a versatile platform not only for studying viral assembly, budding, and entry processes but also for developing novel antiviral strategies targeting those critical steps in SARS-CoV-2 life cycle.

## Results

### Design of VEE-SARS-CoV-2 viral vectors

**Figure 1:**
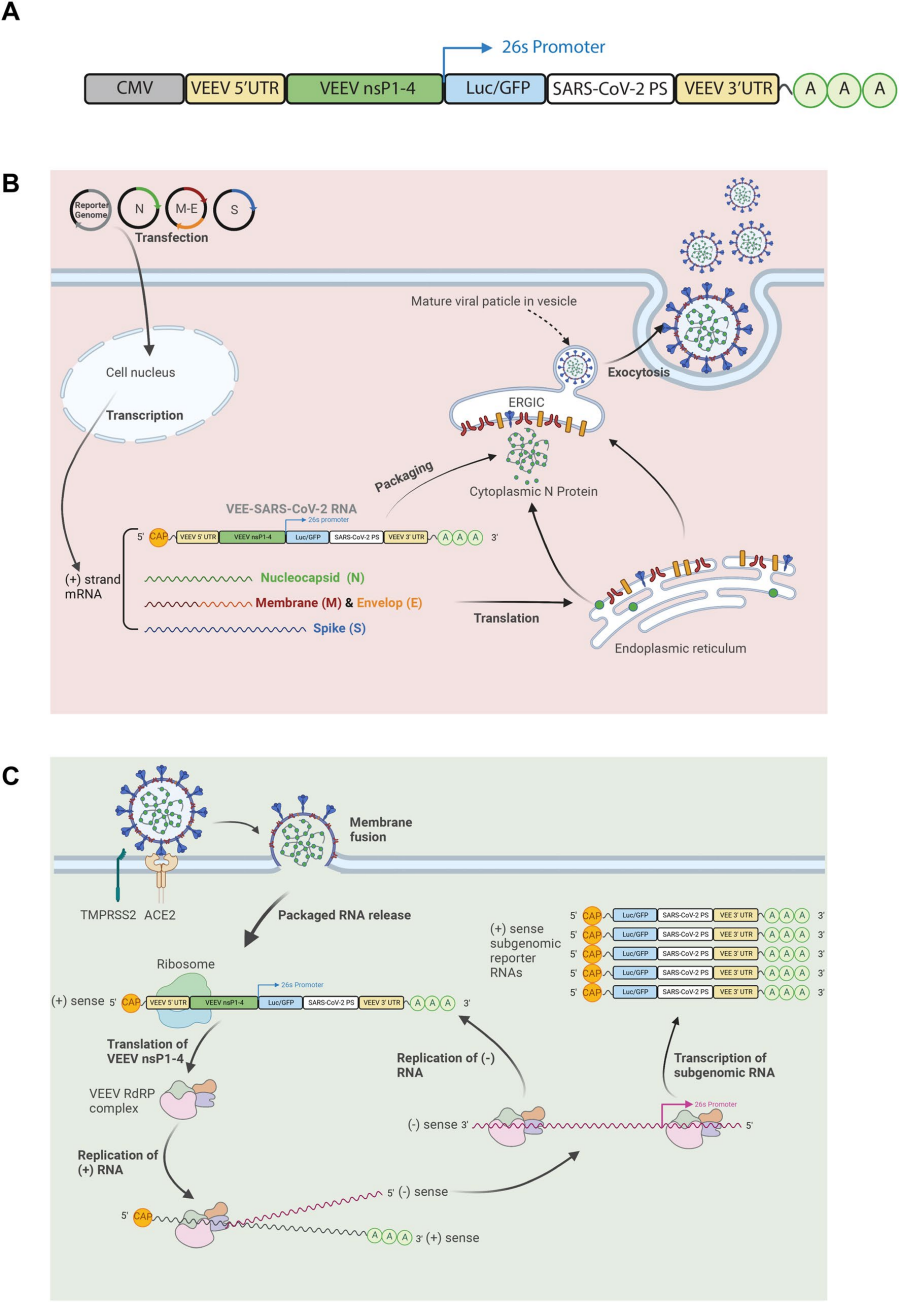
Design of VEE-SARS-CoV-2 viral vectors. (**A**) Schematic diagram of the core elements in VEE-SARS-CoV-2 plasmid including a CMV promoter, 5’ UTR and nsP1-4 gene of VEEV, 26 subgenomic VEEV promoter (26S), reporter genes (Luc or GFP), the SARS-CoV-2 packaging signal (PS9 sequence), 3’ UTR of VEEV genome, and a poly(A) signal. (**B**) Mechanistic illustration of production of VEE-SARS-CoV-2 viral vectors. Components of viral vectors were produced in 293T cells by co-transfection with plasmids carrying structural protein genes (S, M-E, N) and reporter gene with regulatory elements for amplification and expression. The viral vectors are assembled and released from 293T cells. (**C**) Schematic presentation of transduction of viral vectors. RNA amplification and reporter gene expression are accomplished in target cells via ACE2-mediated internalization, translation of VEEV nsP1-4 genes, formation of VEEV nsP1-4 protein complex, and synthesis of the negative-strand RNA. The newly synthesized negative-strand RNA serves as the template for synthesis of positive strand RNA and production of sub-genomic RNA encoding reporter gene.

pcDNA3.1 plasmid vector was employed for construction of the reporter-carrying transfer plasmid (named VEE-Luc/GFP-PS9). As shown in Fig. 1A, the transfer plasmid has a cytomegalovirus (CMV) promoter, the VEEV 5’ untranslated region (UTR), an open reading frame (ORF) of VEEV nsP 1–4 encoding for VEEV RNA replication machinery, a 26 subgenomic promoter to drive downstream gene expression, the SARS-CoV-2 packaging signal sequence (PS9)^18^, and the VEEV 3’ UTR with a poly A tail for RNA stability. Other plasmids carrying SARS Cov-2 structural protein genes are obtained from Addgene.

Figure 1B is a proposed mechanism for generating VEE-SARS-CoV-2 viral vectors in producer cells. The transfer plasmid is co-transfected into HEK293T cells with 3 other plasmids carrying the 4 structural protein genes of SARS-CoV-2 (S, M-E, N), respectively. After transfection, all SARS-CoV-2 structural proteins are produced and translocated into endoplasmic-reticulum-Golgi intermediate compartment (ERGIC) to be assembled into viral particles. Meanwhile, the positive-stranded artificial RNA genome transcribed from the transfer plasmid is selectively packaged into the viral vectors with the help of N protein and PS9 package signal sequence in the RNA transcript. The mature viral particles are then released into the cell culture media via exocytosis.

Figure 1C is a proposed mechanism for VEE-SARS-CoV-2 viral vector-mediated gene delivery and gene expression in target cells. The entry of VEE-SARS-CoV-2 vectors into target cells is initiated by the binding of S protein of VEE-SARS-CoV-2 vectors to ACE2 receptor, primed by TMPRSS2, followed by the release of VEE-SARS-CoV-2 RNA into target cells. The ORF encoding VEEV nsP1-4 on the 5’ end of the positive-sense RNA can be readily translated into proteins to form the VEEV RdRP complex. Subsequently, the RdRP complex synthesizes the negative strand of the introduced RNA. The newly synthesized negative-strand RNA serves as the template for two events: synthesis of the positive-strand RNA, and transcription of the subgenomic RNA encoding the reporter gene, which is driven by the 26 subgenomic (26S) promoter on the negative strand of VEE-SARS-CoV-2 RNA. The transcription process is highly efficient and the copy number of reporter-coding subgenomic RNAs is at least tenfold higher than that of the full-length VEE-SARS-CoV-2 RNA^26^. Notably, the SARS-CoV-2 structural protein genes are encoded by separate plasmids not by the VEE-SARS-CoV-2 RNA. Therefore, this self-amplifying RNA vector transduces target cells in a single-round fashion without synthesizing new progeny of virions.

### Production and optimization of VEE-SARS-CoV-2 vectors

**Figure 2:**
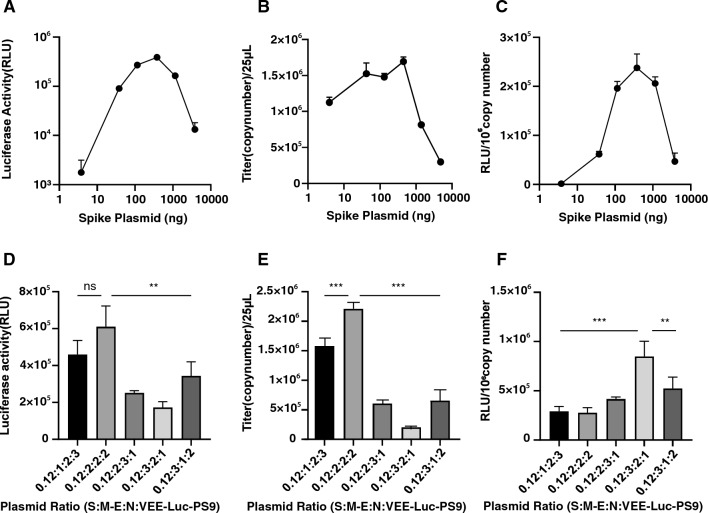
Optimization of VEE-SARS-CoV-2 viral vector production. (**A**) Optimization of the ratio between the plasmid carrying spike protein gene and the rest of plasmids including those carrying M-E, N, and the transfer plasmid for transduction activity. (**B**) Viral vector titer. (**C**) Luciferase activity per 10^6^ genome copies of viral vectors. (**D**) Optimization of transduction activity. (**E**) Titer of viral vectors at different mass ratios of each plasmid with fixed amount of spike plasmid. (**F**) Luciferase activity per 10^6^ genome copies of vectors. Data are presented as the mean ± SD of 3 independent experiments. Statistical significance was determined by one-way ANOVA with Tukey Post Hoc tests. **p < 0.01, ***p < 0.001.

To produce and optimize the yield and transduction activity of VEE-SARS-CoV-2 vectors, we first focused on the ratio between the plasmid carrying spike protein gene and the total of plasmids used in transfection. The ratio impact on the yield of viral vectors was quantified by qPCR-based titration of the genome copy number of viral vectors and luciferase reporter assay in transduced cells. Over a range of 3.8 ng to 3.8 μg of a total 19 μg plasmids used for transfection of 293T cells, 380 ng (2% of total plasmids) of plasmid with spike protein gene generated the highest luciferase activity in ACE2-TMPRSS2-293T cells (Fig. 2A). The qPCR-based titration assay also showed the highest genome copy number of viral vectors at 380 ng of S plasmid in a total of 19 μg plasmids (Fig. 2B), in agreement with the ratio between luciferase gene expression level (defined quantitatively by relative light unit) and the copy number of luciferase RNA copy number of viral vectors (Fig. 2C) Next, we fixed the amount of plasmid with spike protein gene at 380 ng and optimized the ratio of other 3 plasmids (M-E, N, and VEE-Luc-PS9). The results showed that the plasmid mass ratio (S: M-E: N: VEE-Luc-PS9) at 0.12:2:2:2 was optimal (Fig. 2D–F). These data suggest that the SARS-CoV-2 viral vectors require much lower amount of spike protein with respect to other structural proteins, which is consistent with the finding of a previous study^18^.

### Characterization of VEE-SARS-CoV-2 vectors

**Figure 3:**
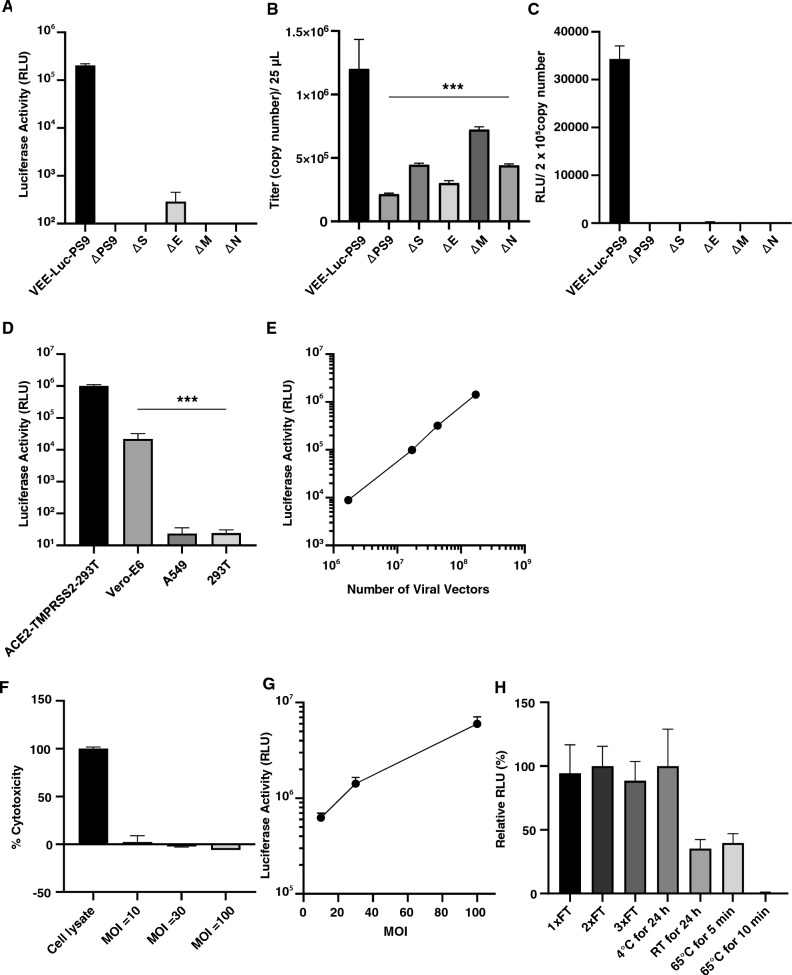
Characterization of VEE-SARS-CoV-2 viral vectors. (**A**–**C**) The effects of structural gene and package signal sequence on transduction activity of VEE-SARS-CoV-2 vectors prepared by co-transfection of 293T cells with all but lacking one structural gene or package signal sequence. The viral vectors were prepared and titrated by qPCR and examined for transduction activity in ACE2-TMPRSS2-293T cells. (**D**) Cell type-dependent transduction activity of VEE-SARS-CoV-2 viral vector. Same number of cells (2 × 10^5^ /well) were transduced with equal number of VEE-SARS-CoV-2-Luc vectors. The transduction activity was determined by luciferase assay at 48 h post-transduction. (**E**) Dose-dependent transduction of VEE-SARS-CoV-2 viral vectors. Same number of ACE2-TMPRSS2-293T cells (2 × 10^5^ /well) were transduced with increasing amount of VEE-SARS-CoV-2-Luc vectors and luciferase activity was determined. (**F**) Cytotoxicity assessment of VEE-SARS-CoV-2-Luc vectors in ACE2-TMPRSS2-293T cells. Lactate dehydrogenase (LDH) activity in the supernatant of transduced cells was determined 48 h post-transduction. Cell lysate was used as the positive control for total release of LDH. (**G**) Luciferase gene expression as function of MOI. (H) VEE-SARS-CoV-2-Luc vector stability under different conditions. All luciferase activity in transduced cells was measured 24 h post-transduction except for (**D**) and (**F**). Data are presented as the mean ± SD of 3 independent experiments. Statistical significance was determined by one-way ANOVA with Tukey Post Hoc tests. ***p < 0.001.

The role of each functional component in VEE-SARS-CoV-2 vectors was examined using a series of viral vectors generated using minus one approach. HEK293T cells were transfected with plasmids withdrawing one of the 4 plasmids carrying structural protein genes or the PS9 packaging signal sequence. Viral vectors were purified separately and transduced into ACE2-TMPRSS2-293T cells. The luciferase assay data show that the viral vectors completely lost their transduction activity when missing any plasmids carrying the structural protein gene (Fig. 3A–C). ΔPS9 viral vectors show no transducing activity and extremely low genome copy number revealed by qPCR titration assay, indicating the packaging activity of VEE-SARS-CoV-2 vector is dependent upon the PS9 packaging signal sequence. In addition, although the moderate copy number of VEE-SARS-CoV-2 genome was detected in viral vectors lacking structural protein genes, lack of luciferase activity suggests that all structural protein genes are required for the vector transduction activity. Overall, these results confirm that all functional components included in the vector design are essential for producing functional VEE-SARS-CoV-2 viral vectors.

We then set to investigate the cellular tropism of VEE-SARS-CoV-2 vector in various cell lines. In addition to ACE2-TMPRSS2-293T cell line, VEE-SARS-CoV-2 vectors also exhibited robust transduction activity in Vero-E6 cells, an African green monkey kidney cell line endogenously expressing ACE2. Not surprisingly, our vectors failed to transduce ACE2-negative cell lines, such as A549 and HEK293T (Fig. 3D). Taken together, VEE-SARS-CoV-2 vectors share the same cellular tropism as the authentic SARS-CoV-2. The ACE2-TMPRSS2-293T cells display the best transduction activity.

The dose-dependent transduction of VEE-SARS-CoV-2 viral vectors was characterized and Fig. 3E shows that the level of luciferase expression was correlated with the number of VEE-SARS-CoV-2 vectors used. This linear dose dependence suggests that VEE-SARS-CoV-2 vectors are suitable for quantitation of antiviral agents including neutralizing antibody and antiviral drugs. More importantly, lactate dehydrogenase assay suggests that three ascending doses (Multiplicity of Infection = 10, 30, 100) did not induce any significant cytotoxicity in transduced cells 48 h post-transduction (Fig. 3F), but increased reporter gene expression (Fig. 3G)**.** The low cytopathic effect of the VEE-SARS-CoV-2 vectors could be attributed to mutations in the 5’ UTR and nsP2/3^27^. Lastly, the stability of VEE-SARS-CoV-2 vectors was tested under different conditions. The results show that the transduction activity remains the same upon 3 × freeze–thaw cycles on ice or storage at 4 °C for 24 h. However, its transduction activity decreased by 65% when kept for 24 h at room temperature. The VEE-SARS-CoV-2 vectors were completely inactivated when kept at 65 °C for 10 min (Fig. 3H).

### Higher level and extended gene expression mediated by VEE-SARS-CoV-2 viral vectors

**Figure 4:**
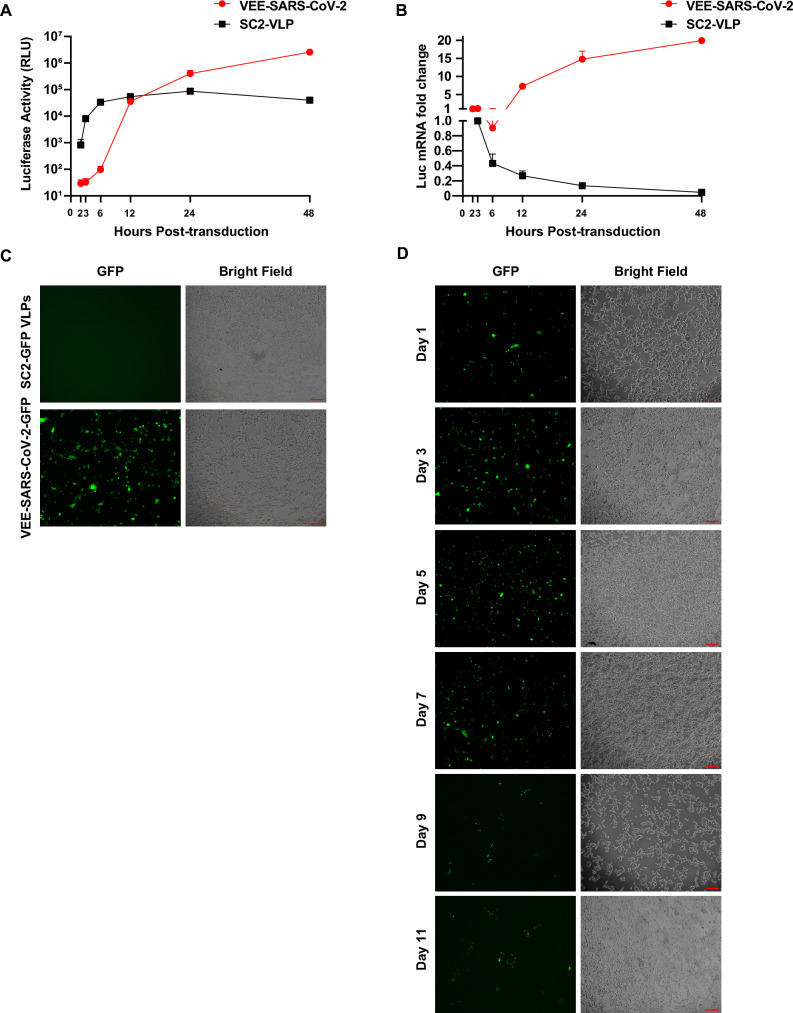
Comparison of time-dependent reporter gene expression in ACE2-TMPRSS2-293T cells transduced by VEE-SARS-CoV-2 vectors and SC2-VLPs. (**A**) Luciferase gene expression level and (**B**) folds of luciferase mRNA change in ACE2-TMPRSS2-293T cells after transduction by VEE-SARS-COV-2-Luc vectors and SC2-Luc-VLP. Transduced cells were harvested at the indicated times, and luciferase assay and real-time qPCR analysis were performed. The fold change was normalized to the first time point determined (2 h post-transduction). GAPDH was used as the internal control. Data are presented as the mean ± SD of independent triplicates. (**C**) GFP expression in ACE2-TMPRSS2-293T cells transduced with VEE-SARS-CoV-2-GFP vectors or SC2-GFP VLPs at 48 h post-transduction. (**D**) Time-dependent GFP expression in ACE2-TMPRSS2-293T cells transduced by VEE-SARS-CoV-2-GFP vectors. Transduced cells were passaged every 4 days and GFP expression was recorded under a fluorescence microscope and cell density was photographed under regular microscope at the indicated times. Scale bar = 200 μm.

Next, we performed a side-by-side comparison between VEE-SARS-CoV-2 vectors and the SARS-CoV-2-based viral like particles (SC2-VLP) previously established^18^. The same reporter RNA copy number of VEE-SARS-CoV-2-Luc and SC2-Luc VLPs were used for transduction. SC2-Luc VLP-transduced cells showed positive luciferase activity (RLU > 10^3^) as early as 2 h post-transduction, the luciferase activity peaked at 24 h (Fig. 4A) and decreased thereafter by half in every 24 h. In comparison, VEE-SARS-CoV-2-Luc vectors exhibited a delayed onset of reporter expression and continued to increase within 48 h post-transduction. The VEE-SARS-CoV-2-Luc vectors generated ~ fivefold (4.0 × 10^5^ vs 8.7 × 10^4^) and ~ 65-fold (2.6 × 10^6^ vs 4.0 × 10^4^) higher luciferase gene expression than SC2-Luc VLPs at 24 and 48 h, respectively. This distinct expression kinetics attributes to the VEEV self-amplification and subsequent translation of reporter RNA sequence by nsP1-4 proteins. This mechanism was further manifested by qPCR analysis measuring the copy number change of luciferase transcript in transduced cells. The results showed that at 48 h post-transduction, the reporter transcript copy number increased by 19-fold in the VEE-SARS-CoV-2-transduced cells, but decreased by 24-fold in SC2-Luc VLP-transduced cells (Fig. 4B).

To prove that VEE-SARS-CoV-2 transduction is not reporter-specific, we also produced VEE-SARS-CoV-2-GFP to deliver GFP into ACE2-TMPRSS2-293T cells. A substantially high number of GFP + cells was observed in target cells transduced by VEE-SARS-CoV-2-GFP vectors, whereas SC2-GFP VLPs failed to generate any visible green cells (Fig. 4C). These data confirmed that VEE-SARS-CoV-2 vector induces significantly higher level of reporter gene expression.

The long-term transgene expression resulting from VEE-SARS-CoV-2 viral vectors was also investigated using GFP reporter. As shown in Fig. 4D, VEE-SARS-CoV-2-GFP transduced cells exhibited an extended gene expression which persists at least 11 days after 2 passages of transduced cells. Taken together, these data demonstrate that VEE-SARS-CoV-2 vectors are highly effective in transduction and capable of generating prolonged transgene expression.

### Inhibition of VEE-SARS-CoV-2-mediated transduction by neutralizing antibodies and anti-SARS-CoV-2 agents

**Figure 5:**
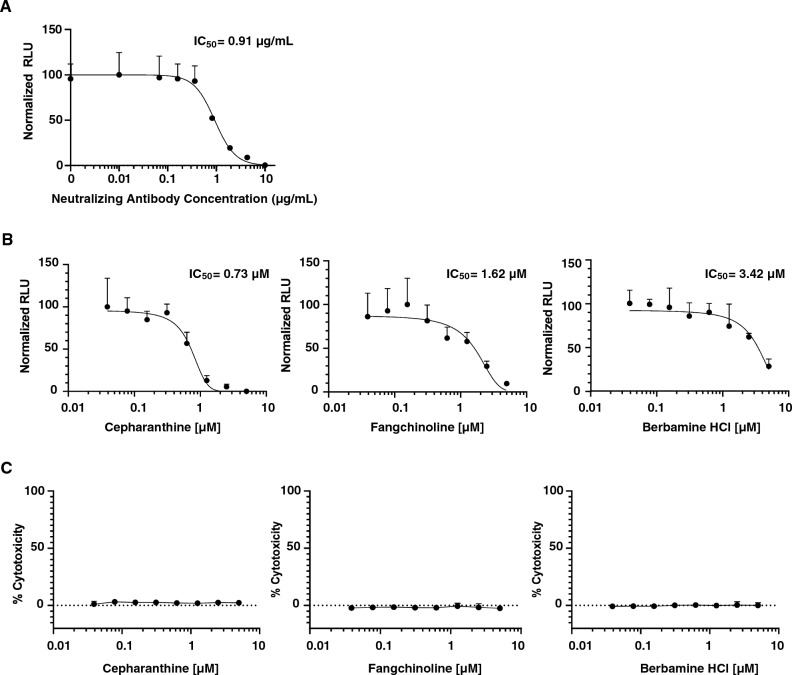
Inhibition of VEE-SARS-CoV-2-Luc-mediated transduction by neutralizing antibodies (**A**) and antiviral agents (**B**). VEE-SARS-CoV-2-Luc vectors were incubated with MM43 SARS-CoV-2 spike neutralizing antibody, antiviral compound of cepharanthine, fangchiboline, or berbamine hydrochloride, for 1 h in a volume of 100 µL at indicated concentration. The mixture was added to ACE2-TMPRSS2-293T cells (2.5 × 10^4^ /well) pre-seeded one day earlier. Transduction proceeded for 3 h, and the medium was replaced with fresh medium. Luciferase activity was determined at 48 h post-transduction. (**C**) Cytotoxicity of compounds at different concentrations was determined at 48 h post-treatment by LDH cytotoxicity assay. Data are presented as the mean ± SD of independent triplicates.

We performed proof-of-concept experiments to demonstrate that VEE-SARS-CoV-2 system can be used for assessing neutralizing antibody against SARS-CoV-2. A dose-dependent curve was obtained from a neutralization assay using VEE-SARS-CoV-2-Luc vector pre-treated with a monoclonal antibody (40591-MM43) against SARS-CoV-2 spike protein (Fig. 5A). Notably, the IC_50_ obtained is 0.91 μg/mL, close to the IC_50_ value of 1.41 μg/mL against spike-pseudotyped lentiviruses provided by the manufacture.

We also used VEE-SARS-CoV-2-Luc vectors to test antiviral activity of compounds, cepharanthine, berbamine hydrochloride, and fangchinoline with reported activity inhibiting SARS-CoV-2 infection. These three compounds were known for their activities in blocking spike protein-mediated viral entry^9,28,29^ and were selected as model drugs to validate the drug testing assay using our VEE-SARS-CoV-2-Luc vectors. All model drugs showed antiviral activities in a dose-dependent manner (Fig. 5B) without inducing cytotoxicity to target cells in the concentration range tested (Fig. 5C). Among these compounds, cepharanthine exhibited the highest activity with an IC_50_ of 0.73 μM, which is close to IC_50_ (0.35 μM^28^ and 1.90 μM^30^) determined by infection assays with authentic SARS-CoV-2 viruses. Consistent with the reported data obtained from SARS-CoV-2^29^, berbamine hydrochloride and fangchinoline showed lower activity against VEE-SARS-CoV-2 vector. Collectively, these data suggest that our novel VEE-SARS-CoV-2-Luc vector is a robust model for evaluating neutralizing antibody and antiviral drugs against SARS-CoV-2.

## Discussion

Experimental procedures involving SARS-CoV-2 virus require BSL-3 laboratories, which hinders the progress of understanding SARS-CoV-2 virology and developing novel antiviral strategies. The VEE-SARS-CoV-2 vectors developed in this study offer an alternative approach with its unique advantages. Firstly, the VEE-SARS-CoV-2 vectors are safe to use in BSL-2 conditions. Since the structural protein genes of SARS-CoV-2 are expressed *in trans*, the VEEV self-amplification machinery produces more copies of reporter gene without synthesizing new virions in transduced cells (Fig. 1B). Secondly, the VEE-SARS-CoV-2 vectors share structural similarities with SARS-CoV-2 and recapitulate the process of viral RNA packaging, viral release, and entry into ACE2 + cells. Compared to pseudotyped viruses bearing only one structural protein (S protein), the VEE-SARS-CoV-2 comprises of all structural proteins and an artificial genome encoding a reporter gene, which enables quantitation of transduced gene expression. The structural resemblance could be utilized to investigate the critical steps in SARS-CoV-2 life cycle such as viral entry, genome packaging, and budding. Thirdly, the VEE-SARS-CoV-2 vectors exhibit high gene delivery efficiency and prolonged gene expression (Fig. 4). The VEE-SARS-CoV-2 viral vectors represent an efficient SARS-CoV-2 substitute system generating significantly more GFP + cells comparing to other viral particle systems^18,31^.

One important application of VEE-SARS-CoV-2 vectors is for drug screening and neutralizing antibody evaluation. Comparing to S-pseudotyped viruses that only contain SARS-CoV-2 S protein instead of all 4 structural viral proteins, the VEE-SARS-CoV-2 vectors are better model viral particles for drug testing. Data shown in Fig. 5 provide direct evidence in support of the utility of the VEE-SARS-CoV-2 system for evaluating neutralizing antibody and small molecules targeting S protein. Further studies may utilize this system for high throughput screening of novel drug candidates targeting E, M, and N proteins.

The transduction activity of VEE-SARS-CoV-2 vector system in animal models remains to be examined. A worthwhile endeavor would be introducing the VEE-SARS-CoV-2 vectors into murine models that are permissive to SARS-CoV-2 infection^32,33^. As a surrogate system for the wild type virus, our system could model the viral tropism and shed insight on infection mechanisms in vivo. The advantages of the VEE-SARS-CoV2 vectors in vaccine development due to its characteristics of prolonged gene expression warrantee further studies. Practically, S-protein gene could be included into VEE-SARS-CoV-2 vectors as a transgene to elevate S-protein expression and immune response against S-protein. Similarly, any of the 4 structural protein gene can be cloned and overexpressed in the presence of all structural proteins for maximal immune stimulation. As a gene delivery vehicle, the potential of VEE-SARS-CoV-2 vectors for gene delivery and gene therapy is another important area of further research. As the VEE-SARS-CoV-2 vectors infect the same target cells as SARS-CoV-2 virus, one can foresee the possibility of delivery of siRNA or CRISPR-based genome editing machinery to target cells of SARS-CoV-2 virus to inhibit viral amplification.

Successful gene delivery of the current vector system appears dependent on a high expression level of ACE2 in target cells. As shown in Fig. 3B, luciferase activity was ~ 50 fold higher in 293T cells overexpressing hACE2 and TMPRSS2 compared to in Vero E6 cells that express lower level of endogenous ACE2. Future studies are needed to investigate the transduction activity of VEE-SARS-CoV-2 in target cells or organs with relatively low level of hACE2. Another limitation of VEE-SARS-CoV-2 as a drug screening system is the absence of the SARS-CoV-2 replication machinery. In contrary to other SARS-CoV-2 model systems such as SARS-CoV-2 replicons^5–8^, trans-complementation system^34,35^, and attenuated SARS-CoV-2 with deletions of accessory genes^36^, VEE-SARS-CoV-2 fails to recapitulate SARS-CoV-2 viral replication. Hence, this system can only be used for screening drugs targeting SARS-CoV-2 structural proteins but not non-structural proteins.

In conclusion, we demonstrate in this study the successful development of novel VEE-SARS-CoV-2 viral vectors consisting of 4 structural proteins of SARS-CoV-2, a self-amplifying machinery, and a transgene. The transducing activity of the vectors is dependent upon ACE2 and all structural proteins and outperforms other systems such as SC2-VLPs^18^. The vectors can be used under BSL-2 conditions as a versatile model system for SARS-CoV-2 virology and antiviral agent development. The vectors developed could be explored for other applications such as vaccine development and gene delivery.

## Materials and methods

### Plasmid construction and preparation

Plasmids encoding SARS-CoV-2 structural protein genes of Wuhan-Hu-1 strain (CoV2-Spike-D614G, CoV2-M-IRES-E, and CoV2-N-P199L) were gifts from Dr. Jennifer Doudna (The University of California, Berkley, Addgene #177960, 177938, 177949). The transfer plasmid VEE-GFP-PS9 was constructed by sequential subcloning of VEE virus replicon from T7-VEE-GFP, a gift from Dr. Steven Dowdy, (Addgene plasmid # 58977^37^) and PS9 sequence from Luc-PS9 (a gift from Dr. Jennifer Doudna, Addgene plasmid #177942) into pcDNA3.1(+) backbone. The first 2141-bp sequence of T7-VEE-GFP was PCR-amplified with Phusion Plus using a primer pair (5’ to 3’): ACGAGCTCTACGACTCACTATAGATGGGCG and CCGGAATTCATGGAAGGGAGGATCC, digested with *SacI* and *EcoRI*, and ligated into pcDNA3.1(+) backbone. Secondly, the *EcoRI* and *NotI* fragment (nt 2136 - 8331) of T7-VEE-GFP plasmid was ligated into the same construct. Thirdly, the VEE 3’UTR sequence (9561–9759 bp) in T7-VEE-GFP plasmid was PCR-amplified by primers (F: CGGCTCGAGGAATTGGCAAGCTGCTTACATAG and R: TGCTCTAGACACATTTCCCCGAAAAGTG), digested with *XhoI* and *XbaI*, and ligated into Luc-PS9. Lastly, the fragment containing PS9-WPRE-VEE 3’UTR generated in the third step was subcloned into the backbone sequence generated in the second step using *NotI* and *PmeI*. For the VEE-Luc-PS9 transfer plasmid, the firefly Luciferase gene was first subcloned to replace the GFP gene in the T7-VEE-GFP plasmid. Then the VEE-Luc-PS9 plasmid was constructed using the same procedure of VEE-GFP-PS9 construction. The extra start codon in the *NdeI* site of T7-VEE-GFP plasmid was mutated by PCR (CATATG to CATATA). A Kozak sequence (GCCACC) was added into the 5’ end of both firefly luciferase and GFP genes in each construct. For VEE-Luc-ΔPS9 or VEE-GFP-ΔPS9 construct, the PS9 sequence was replaced by a scrambled sequence (TTGTAGAAGAAACACATGGCGCGCCTGA) for ligating the plasmid using T4 ligase. The DNA sequences for all plasmids constructed were confirmed by Sanger sequencing and DNA gel electrophoresis. Plasmids used for transfection were prepared using E.Z.N.A. MaxiPrep Kit (Omega Bio-tek).

### Cell culture

HEK293T (a gift from Dr. Houjian Cai, University of Georgia), Vero E6 (a gift from Dr. Marco Archetti, Pennsylvania State University), and A549 (ATCC, CCL-185) cells were cultured in DMEM complete medium supplemented with 10% FBS and 1% penicillin and streptomycin. ACE2-TMPRSS2-HEK293T cells were purchased from InvivoGen and cultured in DMEM with 10% FBS, 1% penicillin and streptomycin, 0.5 μg/mL Puromycin, 200 μg/mL hygromycin, and 100 μg/mL zeocin. Cells are kept in 10 cm petri dishes under 5% CO_2_ in a humidified incubator at 37 °C and passed once in 3 days.

### Preparation of VEE-SARS-CoV-2 viral vectors

HEK293T cells (8 × 10^6^) were seeded in a 10 cm petri dish 24 h before the transfection. For transfection, a total of 19 μg of plasmid DNA containing the desirable plasmids at appropriate plasmid ratio was dissolved in Opti-MEM (Gibco) with a total volume of 500 μL. The plasmid DNA solution was mixed dropwise with 500 μl of PEI prepared by mixing 57 μL of PEI (1 mg/ml) (Sigma-Aldrich) with 443 μL of Opti-MEM. The plasmid/PEI mixture was vortexed for 5 s and incubated at room temperature for 15 min before being added dropwise into each of the 10 cm petri dish of HEK293T cells containing 9 mL of DMEM medium with 10% FBS and 1% penicillin/streptomycin. Transfection mixture was kept with cells overnight and the medium was replaced with fresh medium. At 48 h post-transfection, the culture medium containing viral particles was harvested and centrifuged at 4,000 g for 10 min using Allegra X-15R Centrifuge (Beckman Coulter). The supernatant was harvested and filtered with a 0.45 μm syringe filter and the filtrate was stored at − 80 °C until use. SC2-VLPs were prepared following the same procedure.

### Purification of VEE-SARS-CoV-2 viral vectors

The method of ultracentrifugation was used for purification of viral vectors. The frozen filtrate was thawed on ice and 24 mL were loaded onto 2 mL of 20% sucrose cushion (#84097, Sigma-Aldrich) in a conical bottom tube. The SW32Ti rotor (Beckman Coulter) was used for centrifugation at 28,000 g for 2 h at 4 °C. The supernatant was discarded, and pellet of viral particles was resuspended in 150 μL of PBS solution. The viral vectors were kept on ice and usually used within 1 day after preparation.

### qPCR-based titration of reporter RNA

For determining the viral vector titers, the purified viral vectors (25 μL) were mixed with 500 μL of Trizol solution (Invitrogen) for lysis. The total viral RNA was then extracted following the manufacturer’s instruction. The extracted viral RNA was treated with DNA-free kit (AM1906, Invitrogen), and reverse transcribed using Maxima first strand cDNA synthesis kit (Thermo Scientific). The titres of the VEE-SARS-CoV-2 are defined as reporter RNA copy number/25µL of concentrated viral vector suspension. Usually, the titer of the VEE-SARS-CoV-2 vectors is 1 ~ 2 × 10^6^ copies/25µL. The infectious titer of VEE-SARS-CoV-2 vectors is around 4 × 10^6^ transduction units per milliliter.

In determining the RNA level of luciferase reporter in transduced cells in a 12-well plate, we removed the culture medium at the desirable time after transduction and washed the cells once with PBS. The cells were lysed with 500 μL Trizol solution and the total RNA was extracted according to manufacturer’s instruction. The RNA concentration was determined by NanoDrop Lite Spectrophotometer and 1 μg of RNA was subjected to reverse transcription using Maximal first strand cDNA synthesis kit. The cDNA was then amplified by qPCR and the copy number of luciferase reporter RNA was calculated based on a standard curve established using a tenfold serial dilution of the VEE-Luc-PS9 plasmids. In all cases, the same primer pair (5’ to 3’): GTGGTGTGCAGCGAGAATAG and CGCTCGTTGTAGATGTCGTTAG targeting part of luciferase reporter sequence was used. Real time qPCR was performed in a StepOne Plus Real Time PCR system (Applied Biosystems) using PerfeCTa® SYBR® Green reagent (Quantabio).

### Luciferase assay

A total of 10^5^ ACE2-TMPRSS2-293T cells were seeded in each well of a 12-well plate. After 48 h, the attached cells were transduced with VEE-SARS-CoV-2 viral vectors or SC2-VLPs when needed. The transduction proceeded until the desirable time for analysis of gene expression. Cell culture medium was aspirated, and cells were rinsed with 1 × PBS. The cells were lysed in 100 μL 1 × FLuc-Lysis buffer (Genecopoeia) with 5-min shaking at room temperature. Cell lysates (20 μL) were transferred into a 96-well microplate (#3912, Corning) and mixed with 100 μL of reconstituted luciferase reagent (E1501, Promega). Luciferase activity was immediately measured using CLARIOstar plate reader (BMG Labtech) and presented quantitatively as the relative light unit (RLU).

### Cytotoxicity assay

ACE2-TMPRSS2-293T cells (2.5 × 10^4^ cells/well) in 100 μL DMEM complete medium were seeded in a 96-well plate for 24 h and transduced with VEE-SARS-CoV-2 vectors at desired multiplicity of infection (MOI). Forty-eight hours post-transduction, 50 μL of cell culture medium of transduced cells was transferred into a new 96-well plate and mixed with 50 μL of Pierce LDH Cytotoxicity reaction mixture (Thermo Scientific) at room temperature for 30 min. The reaction was terminated by addition of 50 μL of stop solution. The absorbance was measured at 490 nm and 680 nm, respectively. For positive control, the culture medium of the untreated cells was removed and replaced with the same volume of lysis buffer. Cells were incubated for 5 min to complete cell lysis and 50 μL of cell lysis was used for spectrometric measurement.

### Antibody neutralization assay

A total of 2.5 × 10^4^ ACE2-TMPRSS2-293T cells in 100 μL of DMEM complete medium were seeded in a well of 96-well plate for 24 h. VEE-SARS-CoV-2-Luc viral vectors were incubated with SARS-CoV-2 spike neutralizing antibody (40591-MM43, SinoBiological) at 37 °C for 1 h. The vector-antibody mixture was added to pre-seeded cells and incubated for 48 h. The level of reporter gene expression was determined using the luciferase assay.

### Inhibition of VEE-SARS-CoV-2-Luc vector-mediated transduction by anti-viral agents

Cepharanthine (#T0131), Berbamine dihydrochloride (#T2920), and Fangchinoline (#T3122) were purchased from TargetMol and dissolved in dimethyl sulfoxide (Sigma) with final concentration of 10 mM. ACE2-TMPRSS2-293T cells (2.5 × 10^4^ cells/well) in 100 μL DMEM complete medium were seeded in a 96-well plate. Twenty-four hours later, VEE-SARS-CoV-2-Luc viral vectors pre-incubated for 1 h at 37 °C with each compound at desirable concentrations were added to the pre-seeded cells. The cells were cultured for 3 h, then the mixture was removed and replaced with fresh DMEM complete medium. Luciferase assay was performed 48 h post-transduction. At 48 h post-treatment, cytotoxicity assay was performed using Pierce LDH Cytotoxicity reaction mixture (Thermo Scientific) as described above to determine the cytotoxicity of anti-viral compounds.

### Statistical analysis

Transduction and luciferase assays were performed in triplicates in all experiments and RLU value was presented as mean ± standard deviation. Statistical analysis was performed using one-way ANOVA with Tukey Post Hoc tests by GraphPad Prism 8. GraphPad Prism 8 was also used to plot all the figures and calculate IC_50_ values for assessing neutralizing antibody and antiviral drugs.

## Data Availability

All study data are available in the article. DNA sequence information of plasmids constructed in this study is available in the GenBank repository (accession numbers: OQ749644-OQ749646).
